# Argon Plasma-Assisted Liposuction for Arm Contouring: Safety, Efficacy, and Skin Tightening Outcomes

**DOI:** 10.1093/asjof/ojaf058

**Published:** 2025-06-16

**Authors:** Jesús Benito-Ruiz

## Abstract

**Background:**

Brachioplasty remains the gold standard for addressing excess skin on the arms. However, because of visible scarring and potential complications, new technologies have been explored to enhance skin tightening in combination with liposuction.

**Objectives:**

The authors of this study evaluate the safety and efficacy of argon plasma-assisted liposuction (APAL) for upper-arm contouring by quantifying changes in ptosis reduction, arm diameter, and skin elasticity.

**Methods:**

Fifteen patients (30 arms) were included in the study. All patients underwent power-assisted liposuction combined with argon plasma at 50 W for 3 min per arm. Follow-ups were conducted at 10, 30, and 180 days postprocedure. Cutaneous temperature was measured before treatment and at 1 and 5 min after argon plasma application. The variables assessed at the maximal point of convexity of the abducted arm included thickness (measured through ultrasound), ptosis, diameter (measured using a 3-dimensional camera), and elasticity (measured with a Cutometer). Patient satisfaction was evaluated using the ARM-Q test.

**Results:**

Compared with preoperative levels, there was a significant reduction in all parameters (ptosis, diameter, thickness, and skin elasticity) at each follow-up point. Results stabilized from Day 30 onward. By the end of the study, the mean reduction was 29% for ptosis, 15% for diameter, and 42.7% for thickness. Patient satisfaction (ARM-Q) was notably high, with an average increase from 1.6 to 3 (85%). No major complications were observed, and skin heating remained within a safe threshold.

**Conclusions:**

APAL may be a safe and effective technique for upper-arm contouring, particularly in patients with mild-to-moderate skin laxity.

**Level of Evidence: 4 (Therapeutic):**

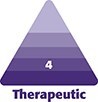

Upper-arm contouring has traditionally posed a challenge in aesthetic surgery because of skin laxity, excess fat deposition, and the natural loss of elasticity with aging. The pendulous arm is a common complaint in middle-aged women. Although brachioplasty (arm lift) remains the gold standard for correcting severe skin redundancy, it has significant drawbacks, including visible scarring, a high revision rate, prolonged recovery, and potential complications, such as seroma, wound infection, and poor wound healing.^1-3^ These drawbacks are difficult to accept, which explains why the last International Society of Aesthetic Plastic Surgery survey showed that this procedure accounted for only 1.5% of the total number of surgical procedures.^4^

As a result, there has been a growing interest in minimally invasive alternatives that can achieve satisfactory contouring without the need for extensive incisions.

Liposuction has long been the primary minimally invasive approach for addressing excess adiposity in the upper arms. However, traditional liposuction alone is limited in its ability to induce skin contraction, particularly in cases where skin elasticity is compromised. This limitation has led to the integration of energy-based devices that enhance postliposuction skin tightening, thereby improving overall aesthetic outcomes.^5^

Over the past decade, several energy-based technologies have been introduced to complement liposuction by stimulating dermal contraction and collagen remodeling, such as the following: radiofrequency-assisted liposuction (RFAL)—technologies such as BodyTite (Invasix Corp., Yokneam, Israel) deliver controlled thermal energy to the subdermal tissue, promoting fat coagulation and skin contraction; laser-assisted liposuction—devices that utilize laser energy to heat collagen fibers, triggering neocollagenesis; ultrasound-assisted liposuction—systems such as VASER (Solta Medical, Bothell, WA) utilize ultrasound waves to emulsify fat while preserving fibrous tissue integrity, leading to better retraction; helium plasma—more recently, plasma-driven energy devices have gained attention for their ability to deliver precise thermal energy to the connective tissue matrix, inducing immediate and sustained skin contraction.^6-9^

Argon plasma technology utilizes argon gas instead of helium and allows for controlled coagulation with minimal tissue penetration, typically limited to a depth of 2 to 3 mm, reducing the risk of perforation and promoting uniform treatment of superficial lesions. Introduced in open surgery during the late 1970s, argon plasma was adapted for endoscopic applications in 1991, significantly enhancing its utility in minimally invasive procedures. In surgical contexts, it has been employed for hemostasis, tissue devitalization, and tumor debulking, offering a noncontact means to coagulate bleeding surfaces and ablate abnormal tissues effectively. In the field of gastroenterology, it has been widely adopted for managing gastrointestinal bleeding from lesions, such as angiodysplasias, gastric antral vascular ectasia, and radiation proctitis. The noncontact nature of argon plasma allows for safe coagulation of lesions in areas with thin walls, such as the cecum, minimizing the risk of perforation.^10,11^

This is a study on Argo Plasma (EMED, Opacz-Kolonia, Poland) to enhance soft-tissue contraction and skin tightening following liposuction in body contouring.

The authors of this study aimed to evaluate the safety and efficacy of argon plasma-assisted liposuction (APAL) for upper-arm contouring, with a focus on the following:

- Quantifying changes in ptosis reduction, arm diameter, and skin elasticity
- Analyzing the safety profile and potential complications.

## METHODS

This prospective study aimed to evaluate the safety and efficacy of our standard protocol combining liposuction with argon plasma (EMED). Fifteen consecutive patients (30 arms) presenting with upper-arm laxity were included. Exclusion criteria were a BMI >30 or a history of previous surgical treatments, including liposuction, on the arms. Informed consent was obtained from all participants, and the study was conducted in accordance with the principles of the Declaration of Helsinki. IRB approval was not required for this study.

For the evaluation of the impact of the reduction in volume and retraction of the skin, measurements in millimeters were made at the point of maximal convexity with the arm at 90°. In addition to the demographic parameters (age and BMI) and surgical information (extracted volume and cutaneous temperature), we evaluated the following:

- Thickness of the subcutaneous tissue (Th: thickness) with ultrasonography (Alpinion XCube I9, Seoul, Korea)
- Diameter of the arm (D: diameter), measured from 3-dimensional (3D) camera images (Artec, Luxembourg)
- Ptosis, measured from 3D camera images as the distance between the lower part of the arm in abduction to a line drawn from the medial epicondyle to the mid-axilla (BF-Edge). The arms were classified following the El-Khatib classification (Supplemental Table).^12^

Elasticity measurements (R0, R2, R5, and R7) through a Cutometer MPA 580 (Courage + Khazaka electronic GmbH, Köln, Germany) with a 6 mm diameter probe. R0 measures the skin’s distensibility and firmness, so lower values indicate greater firmness. R2 is the ratio of elasticity between the immediate elastic recovery and the maximum deformation. R5 refers to the net elasticity, which is the ratio between elastic recovery and maximum deformation. Finally, R7 is the biological recovery or elastic recovery with respect to net deformation.

Patient satisfaction was measured with ARM-Q questionnaire (Memorial Sloan Kettering Cancer Center, NY).

The evaluations were conducted as follows:

- Preoperative, 10, 30, and 180 days: conventional and 3D camera photographs, ultrasound measurements of subcutaneous tissue thickness, and elasticity measurements
- Preoperative and at 180 days: subjective evaluation by the patient through the ARM-Q questionnaire
- Surgery: measurement of the total volume of liposuctioned fat and extracted fat. Skin temperature was measured with an infrared thermometer before the start of the argon plasma treatment, at the end of the treatment, and 5 min after the treatment.

### Surgical Procedure

All interventions were performed under general anesthesia. Infiltration was carried out through the use of a saline solution with 1/1,000,000 epinephrine at a 1:1 ratio. Arm liposuction was performed through PAL (MicroAire, Charlottesville, VA) on 3 quarters of the arm's circumference, except for the inner aspect. Incision for access was made above the olecranon and on the anterior aspect of the axillary fold. After liposuction was complete, argon plasma treatment was applied at 50 W for 3 min in the liposuctioned area. The cannula is withdrawn slowly to allow the ionized current to effectively target the fibrous septa. It is maneuvered in a fan-like pattern, with care taken to avoid overheating the tissue closest to the access site. Although there is no fixed number of passes, the surgeon aims to make multiple passes to ensure comprehensive coverage of the treatment area (Video).

During the first postoperative month, patients wore compression sleeves for 24 h a day and underwent weekly lymphatic drainage sessions. For the 6 months of the study, they were not allowed to undergo any additional treatments (such as radiofrequency) but were permitted to engage in normal physical activity.

### Statistical Analysis

Statistical analyses were conducted through MedCalc Statistical Software version 23.1.5 (MedCalc Software Ltd, Ostend, Belgium). Descriptive statistics, including the mean ± SD, median (interquartile range), mean ± standard error of the mean, and frequency (percentage), were utilized to summarize the demographic data and morphometric characteristics of the arms.

The Shapiro-Wilk test was applied to assess the normality of variable distributions. Because the BF-Edge of the arm, arm diameter, ultrasound thickness measurements, and Cutometer (Courage + Khazaka electronic GmbH) parameters (R0, R2, R5, and R7) followed a normal distribution, repeated-measures analysis of variance was used to evaluate changes in these parameters over time. Post hoc pairwise comparisons were performed through Scheffé's method, with the Greenhouse–Geisser correction applied to account for deviations from sphericity.

A regression analysis was conducted to evaluate the relationships between the volume of fat removed during liposuction, preoperative ultrasound thickness, and preoperative BF-Edge of the arm with the changes in arm diameter, ultrasound thickness, and BF-Edge from the preoperative period to 6 months postoperatively, respectively. Additionally, the associations between preoperative Cutometer (Courage + Khazaka electronic GmbH) parameters (R0, R2, R5, and R7) and changes in BF-Edge over the same period were also assessed.

In addition, multiple regression analysis through the backward elimination method was performed to evaluate the influence of preoperative variables on changes in arm diameter, BF-Edge, and ultrasound thickness from the preoperative period to postoperative Month 6. The predictor variables included age, arm diameter, ultrasound distance, BF-Edge, distance, volume of fat removed during liposuction, temperature, and Cutometer (Courage + Khazaka electronic GmbH) parameters (R0, R2, R5, and R7).

The χ^2^ test was used to assess changes in ARM-Q questionnaire responses between the preoperative period and postoperative Month 6.

A *P*-value of <.05 was considered significant.

## RESULTS

Table 1 summarizes the demographic and surgical data for all patients. All patients were women, with a mean age of 52 years (38-66 years). The mean BMI was 24.5 (19.3-29.7). Twenty-eight arms were classified as El-Khatib 2B, and 2 were classified as 2A (Supplemental Table).

**Table 1.** Demographic Data of the Series

	*n*	Minimum	Maximum	Mean	SD	95% CI	Median	95% CI	25-75 P	Normal distr.
Age	15	38	66	52.33	8.69	49.08-55.58	56	48.52-58.00	45.00-58.00	0.0032
Volume (cc)	30	125	550	348.33	111.31	306.76-389.89	350	300-375	300-425	0.3853
BF-Edge (mm)	30	38.400	80.000	61.81	9.53	58.25-65.37	62.90	58.30-65.25	57.00-68.05	0.3241
Diameter (mm)	30	286.200	390.000	337.79	26.80	327.78-347.80	333.70	320.27-347.70	320.00-356.40	0.3119
Thickness (mm)	30	12.900	37.200	22.25	7.49	19.45-25.05	19.75	18.00-24.05	17.40-25.40	0.0017
R0	30	0.190	1.076	0.58	0.23	0.49-0.66	0.52	0.44-0.67	0.42-0.70	0.2924
R2	30	65.700	96.300	84.15	9.60	80.57-87.74	87.40	78.14-90.65	76.50-93.20	0.0180
R5	30	51.800	184.200	117.54	33.32	105.10-129.99	126.70	98.76-134.40	90.70-139.70	0.5965
R7	30	42.100	90.800	73.82	12.88	69.00-78.62	76.35	72.37-81.59	68.10-82.90	0.0304

CI, confidence interval; SD, standard deviation.

The mean volume aspirated was 348.30 cc (125-550 cc). The mean preoperative BF-Edge distance was 55.43 mm (38.4-80 mm), and the mean diameter was 337.79 mm (286.2-390 mm). The mean preoperative ultrasound thickness was 22.25 mm (12.9-37.2 mm).

The mean preoperative R0 was 0.57 (0.19-1.07), the R2 was 84.15 (65.7-96.3), the R5 was 117.54 (51.8-184.2), and the R7 was 73.82 (42.1-90.8).

### Temperature

**Figure 1.**
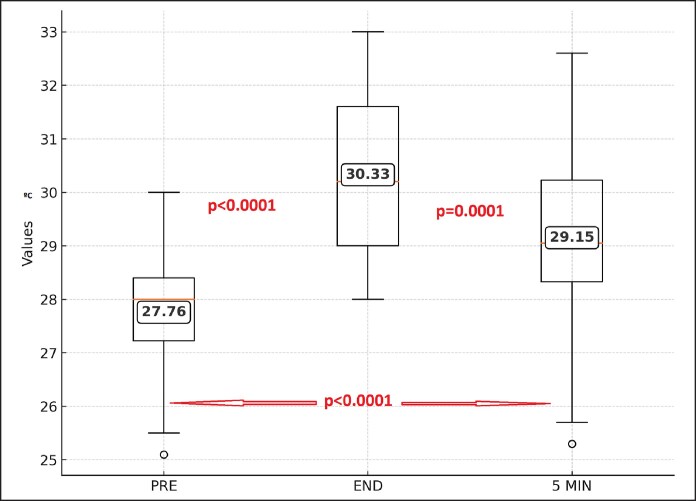
Cutaneous temperature with argon plasma treatment just before the treatment, at the end of the procedure, and 5 min after finishing.

Figure 1 depicts the cutaneous temperature. There was a statistically significant difference between the peoperative values (mean 27.76°C, 25-30°C) and the measurements at the end (mean 30.33°C, 28-33°C) and at 5 min after the procedure (mean 29.15°C, 25.30-32.60°C; *P* < .0001) and between the measurements at the end and at 5 min after the procedure (*P* = .0001). This means that even when the temperature is close to the preoperative value, the heat still dissipates and continues to reduce after this 5 min period.

### Ptosis: BF-Edge Distance

The mean preoperative distances were 55.43 mm (41.5-73.5 mm) and 42.32 mm (32.34-49.7 mm) at 10 days, 39.9 mm (28.69-49.04 mm) at 30 days, and 38.36 mm (19-53 mm) at 180 days.

**Figure 2.**
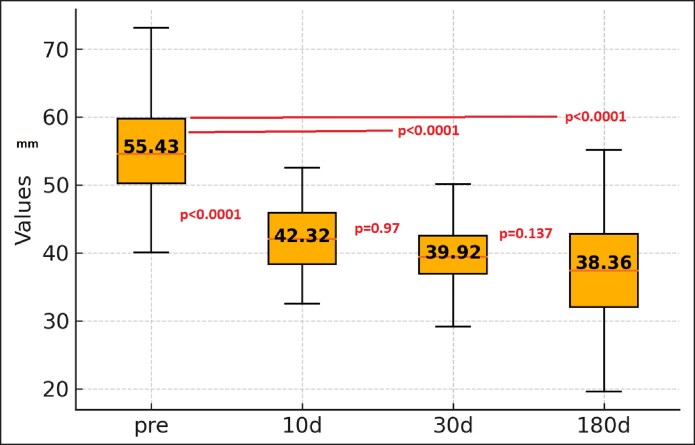
BF-Edge distance at the point of maximal convexity with the arm abducted at 90°, which reflects ptosis and improvement over time.

Compared with preoperative levels, there was a significant reduction in the distance from the bicipital canal edge to the free arm edge at all measurement points (*P* < .0001). There was a slight statistically significant difference (*P* = .012) between 180 and 10 days and no statistically significant difference between 10 and 30 days (*P* = .97) or 180 and 30 days (*P* = .137; Figure 2).

### Diameter

The mean preoperative diameter was 337.79 mm (286.2-390 mm). At 10 days, it was 304.36 mm (261.25-341.93 mm); at 30 days, it was 295.07 mm (235-326.2 mm); and at 180 days, it was 290 mm (220-346.11 mm).

**Figure 3.**
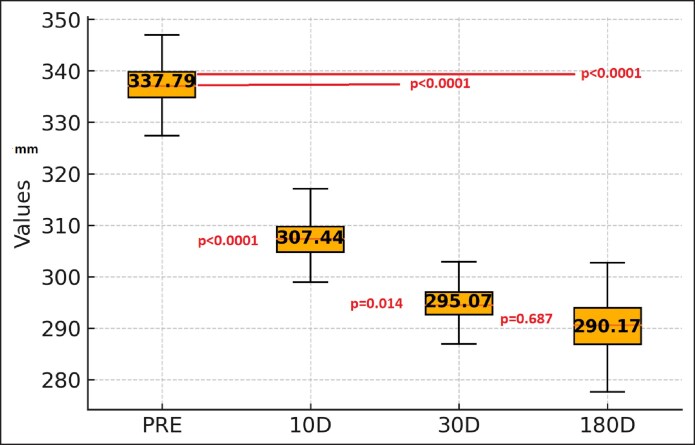
Diameter of the arm at the point of maximal convexity with the arm abducted at 90°.

Compared with the preoperative values, there was a significant reduction in the diameter of the arm at all measurement points (*P* < .0001). There was a slight statistically significant difference between 10 and 30 days (*P* = .014) and 10 and 180 days (*P* = .023) but no difference between 30 and 180 days (*P* = .687; Figure 3).

### Thickness

The preoperative mean was 22.25 mm (12.9-37.2 mm). After 10 days, the mean thickness was 15.12 mm (8.7-22.7 mm); at 30 days, it was 14.29 mm (9-23.7 mm); and at 180 days, it was 12.84 mm (0.04-0.20 mm).

**Figure 4.**
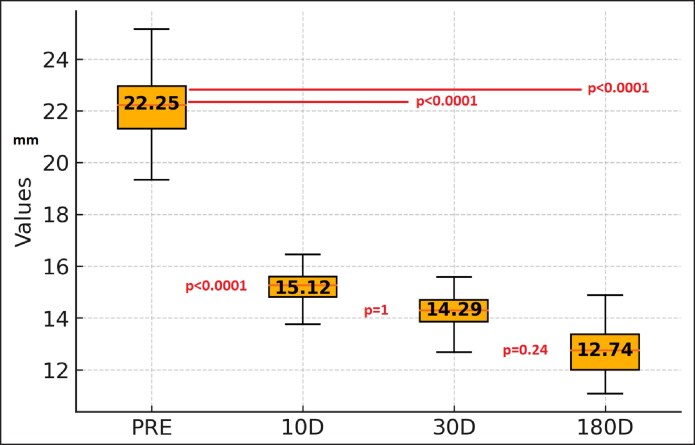
Thickness at the point of maximal convexity with the arm abducted at 90°.

Compared with preoperative levels, there was a significant reduction in tissue thickness, measured by ultrasound, at all measurement points (*P* < .0001). The difference between 10 and 180 days was slightly significant (*P* = .047) but no difference between 10 and 30 days and 30 and 180 days (*P* = 1 and *P* = .24, respectively; Figure 4).

### Skin Elasticity

#### R0 (Skin Distensibility/Firmness)

The preoperative mean R0 was 0.57 (0.19-1.07). At 10 days, the mean was 0.55 (0.1-1.03); at 30 days, it was 0.36 (0.18-0.77); and at 180 days, it was 0.36 (0.23-0.63).

Compared with the preoperative values, there was a significant reduction in the Cutometer (Courage + Khazaka electronic GmbH) R0 values at Days 30 and 180 (*P* = .0005 and *P* < .0001, respectively), but not with Day 10 (*P* = 1). Compared with the measurements taken on Day 10, the measurements taken on Days 30 and 180 were significantly lower than those taken on Day 10 (*P* = .0013 and *P* = .0003, respectively).

#### R2 (Ratio of Elasticity)

The preoperative mean value was 84.15 (65.7-96.3). After 10 days, the mean was 76.17 (29.2-96.5); at 30 days, the value was 65.19 (26.4-92.1); and at 180 days, it was 62.5 (33-86.2).

Compared with the preoperative values, there was a significant reduction in the Cutometer (Courage + Khazaka electronic GmbH) R2 values at all measurement points (*P* = .01 between 10 days and the preoperative values, *P* < .0001 between the preoperative value and 30 days, and between the preoperative value and 180 days). There was no difference between the measurements at 10 and 30 days (*P* = .1). The measurement on Day 180 was significantly lower than that on Day 10 (*P* = .0004) but not with 30 days of measurement (*P* = .1).

#### R5 (Net Elasticity)

The mean preoperative value for R5 was 117.54 (51.8-184.2). At 10 days, it was 141.57 (20-312.2); at 30 days, it was 80.21 (15.5-182.3); and at 180 days, it was 65.2 (27.5-103.1).

Compared with the preoperative values, there was a significant reduction in the cutoff R5 values at Days 30 and 180 (*P* = .0009 and *P* < .0001, respectively) but not with 10 days (*P* = .19). Compared with the measurements taken on Day 10, the measurements taken on Days 30 and 180 were significantly lower than those taken on Day 10 (*P* = .0014 and *P* < .0001). There was as well a statistical difference between 10 and 30 days (*P* = .0014) and no difference between 30 and 180 days (*P* = .37).

#### R7 (Biological Recovery)

The mean preoperative value for R7 was 73.82 (42.1-90.8). At 10 days, the mean was 63.65 (15.7-85.4); at 30 days, the mean was 50.74 (13-81.4); and at 180 days, the mean was 48.56 (24.9-73.1).

Compared with the preoperative values, there was a significant reduction in the Cutometer (Courage + Khazaka electronic GmbH) R7 values at all measurement points (*P* = .0013 at 10 days and *P* > .0001 at 30 and 180 days). Compared with the measurements taken on Day 10, the measurements taken on Days 30 and 180 were significantly lower than those taken on Day 10 (*P* = .004 and *P* = .0002, respectively). There was no difference between values at 10 vs 30 days and 30 vs 180 days (*P* = .38 and *P* = 1, respectively).

### ARM-Q

**Figure 5.**
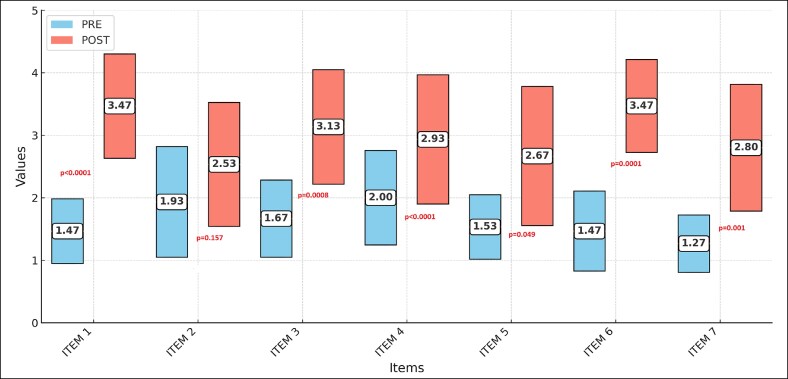
Arm-Q. Scores from 1 to 4, 1 being highly dissatisfied and 4 being highly satisfied. The patients are asked about how much satisfied or dissatisfied they are with: Item 1: your upper arms size; Item 2: how smooth your upper arms look?; Item 3: the shape of your upper arms; Item 4: how the skin of your upper arms look?; Item 5: How toned your upper arms look?; Item 6: How your upper arms look when lifted them up?; Item 7: How your upper arms look when they are not covered?

**Figure 6.**
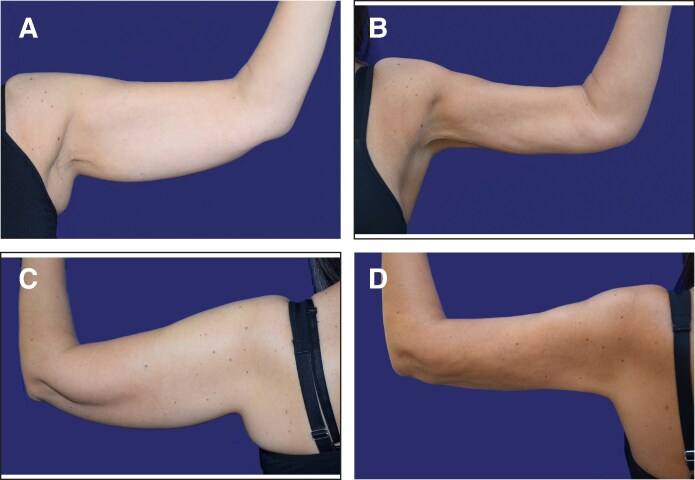
A 48-year-old female patient. Left arm. El-Khatib 2b. The extracted volume was 250 cc. Preoperative BF-Edge distance of 57 mm. Postoperative distance at 6 m 49.6 mm. Preoperative thickness: 22.4 mm; postoperative thickness: 180 mm. (A) Preoperative, front. (B) Postoperative at 6 m, front. (C) Preoperative, back. (D) Postperative, back, at 6 m.

**Figure 7.**
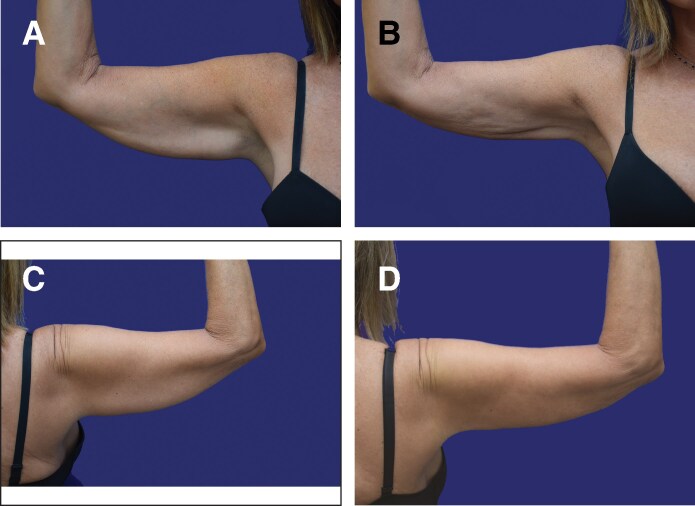
A 60-year-old female patient. Right arm. El-Khatib 2b. Extracted volume of 375 cc. Preoperative BF-Edge distance of 51 mm. Postoperative distance at 6 m 34.5 mm. Preoperative thickness: 28.2 mm; postoperative thickness: 13.5 mm. (A) Preoperative, front. (B) Postoperative at 6 m, front. (C) Preoperative, back. (D) Postoperative, back, at 6 m.

**Figure 8.**
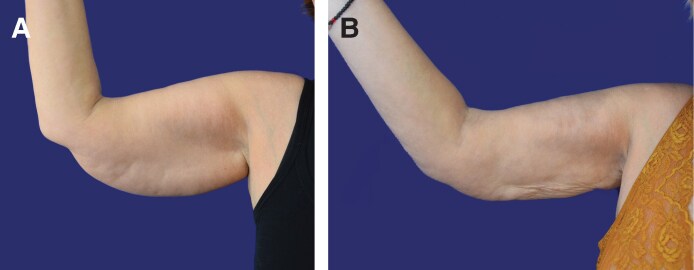
Fifty-nine-year-old female patient. Right arm. El-Khatib 2b. Extracted volume of 500 cc. Preoperative BF-Edge distance of 65.8 mm. Postoperative distance at 6 m 34.5 mm. Preoperative thickness: 37.6 mm; postoperative thickness: 9 mm. (A) Preoperative, front. (B) Postoperative at 6 m, front. Some wrinkling because of deflation is observed.

All postoperative values at 180 days were significantly better than the preoperative values (Figure 5). Figures 6-8 show some results from the procedure. The responses measuring how satisfied or dissatisfied they were (Table 2):

1. The size of your upper arms. The mean preoperative value was 1.47 and the mean postoperative value was 3.47 (*P* < .0001).
2. How smooth your upper arms look? The mean preoperative value was 1.93, and the mean postoperative value was 2.53 (*P* = .157).
3. The shape of your upper arms? The mean preoperative value was 1.67, and the mean postoperative value was 3.13 (*P* = .0008).
4. How the skin of your upper arms look? The mean preoperative value was 2, and the mean postoperative value was 2.93 (*P* < .0001).
5. How toned your upper arms look? The mean preoperative value was 1.53, and the mean postoperative value was 2.67 (*P* = .049).
6. How your upper arms look when you lift them up? The mean preoperative value was 1.47, and the mean postoperative value was 3.47 (*P* = .0001).
7. How your upper arms look when they are not covered (eg, in a sleeveless shirt). The mean preoperative value was 1.27, and the mean postoperative value was 2.80 (*P* = .001).

**Table 2.** ARM-Q Questionnaire

Score	1		2		3		4		*P*-value
	Pre	Post	Pre	Post	Pre	Post	Pre	Post	
Arm size	8 (53.3)	1 (6.7)	7 (46.7)	0 (0.0)	0 (0.0)	5 (33.3)	0 (0.0)	9 (60.0)	<.0001*
How smooth (no irregularities) do the arms look?	5 (33.3)	3 (20.0)	7 (46.7)	3 (20.0)	2 (13.3)	7 (46.7)	1 (6.7)	2 (13.3)	.1570
Arm shape	6 (40.0)	1 (6.7)	8 (53.3)	2 (13.3)	1 (6.7)	6 (40.0)	0 (0.0)	6 (40.0)	.0008
Skin appearance of the arms?	4 (26.7)	2 (13.3)	7 (46.7)	2 (13.3)	4 (26.7)	6 (40.0)	0 (0.0)	5 (33.3)	<.0001*
How much toned?	7 (46.7)	3 (20.0)	8 (53.3)	3 (20.0)	0 (0.0)	5 (33.3)	0 (0.0)	4 (26.7)	.0049*
What do they look like when you raise your arms?	9 (60.0)	0 (0.0)	5 (33.3)	2 (13.3)	1 (6.7)	4 (26.7)	0 (0.0)	9 (60.0)	.0001*
What do your arms look like when they are raised and exposed (naked, with short sleeves)?	11 (73.3)	2 (13.3)	4 (26.7)	3 (20.0)	0 (0.0)	6 (40.0)	0 (0.0)	4 (26.7)	.001*

The patients are asked to rate how satisfied or dissatisfied they are with the different items, with 1 meaning very dissatisfied and 4 meaning highly satisfied. * values statistically significant at *P* < .05

### Influence of the Studied Variables on the Outcomes (Regression Analysis)

#### Aspirated Volume of Fat

The volume of extracted fat did not influence the difference in the diameter of the arm between the preoperative period and 180 days. It also did not affect the difference in the BF-Edge distance (ptosis) between the preoperative period and 180 days.

However, a significant relationship was observed between the volume extracted at surgery and the reduction in thickness at 6 months after treatment. In quantitative terms, for each additional cubic millimeter (mm^3^) of volume removed, there was a 0.01537 mm increase in thickness reduction at 6 months after treatment (*P* = .0126).

#### Preoperative Thickness

A significant relationship was observed between the preoperative thickness and arm diameter reduction at 6 months after treatment. In quantitative terms, for each additional millimeter of mean preoperative thickness, there was a mean increase of 1.91 cm in diameter reduction at 6 months after treatment (*P* = .0017).

A significant relationship was observed between the preoperative thickness and the thickness at 180 days. In quantitative terms, for each additional millimeter of mean preoperative thickness, there was a mean increase of 0.7963 mm in thickness reduction at 6 months after treatment (*P* < .0001).

No statistically significant differences in preoperative thickness and the difference of ptosis (BD-Edge distance) were detected.

#### Distance BF-Edge (Ptosis)

There was no relationship between the preoperative BF-Edge distance (ptosis) and the difference in diameter or thickness.

However, a significant relationship was observed between the preoperative free biceps-edge distance and the reduction in the free biceps-edge distance at 6 months after treatment. In quantitative terms, for each additional millimeter of preoperative free biceps-edge distance, there was an increase of 0.7683 mm in the reduction of the free biceps-edge distance at 6 months after treatment (*P* < .0001). In other words, the more ptotic, the better the shrinking response.

#### Preoperative Skin Elasticity (R0, R2, R5, and R7)

There was no relationship between the preoperative R0 and R2 values and the difference in ptosis between 180 days and preoperatively.

Although the preoperative values of R5 (net elasticity: the ratio between elastic recovery and maximum deformation) did not statistically influence the difference in ptosis, a certain trend was observed. For each point increase in the preoperative value of parameter R5, there was a mean decrease of 0.1092 mm in the reduction in the biceps-free edge distance at 6 months after treatment (*P* = .0680). The higher the preoperative R5 was, the better the shrinking response.

A similar phenomenon was observed with R7 (biological recovery: elastic recovery with respect to net deformation). For each point increase in the preoperative value of parameter R7, there was a mean decrease of 0.3176 mm in the free biceps-edge distance at 6 months after treatment (*P* = .0385).

### Complications

There were no major complications. Minor seromas (mean 15.4 cc, 10-20 cc) were identified through ultrasound in 5 arms (16.7%). In 1 patient, the seroma persisted for 1 month. At 6 months, 1 arm in 1 patient displayed visible subcutaneous fibrosis. Patients complained of mild dysesthesia during the first month. Seromas were treated using ultrasound-guided aspiration.

## DISCUSSION

To our knowledge, this is the first study to evaluate the safety and efficacy of APAL in the arms, with a focus on key outcomes such as ptosis reduction, diameter reduction, skin elasticity, and safety profile. The results demonstrate that the procedure provides significant and sustained improvements in arm contour with minimal complications, making it a promising approach for upper-arm reshaping. Other authors have reported good results with helium-plasma technology, but evaluations of improvements have been based mainly on photographs evaluated by surgeons.^13-15^

One of the primary outcomes analyzed was ptosis reduction, measured by the BF-Edge distance. This is a key measurement for evaluating skin retraction. A statistically significant reduction was observed at all postoperative time points (*P* < .0001), with notable and early improvement at 10 days, followed by stabilization at 30 days and beyond. Chia et al compared RFAL (38 W; temperature cutoff 38-40°C) with aggressive subdermal liposuction and reported a reduction in the measured area of 13% vs 8%.^16^ Duncan reported a 50% reduction in the vertical height of pendulous arm skin.^17^ In our study, there was a 29% reduction by the end of the study period.

Similarly, the arm diameter showed a consistent decrease, with significant differences at each postoperative assessment (*P* < .0001). The diameter stabilized between 30 and 180 days, indicating that the maximal tightening effect was achieved within the first month. Importantly, tissue thickness, as assessed by ultrasound, progressively and sustainably decreased.

All the parameters (diameter, BF-Edge distance, and thickness) demonstrated dramatic improvement in the first month, followed by steady and slight further improvement. Stabilization was observed after 30 days. Although it could be argued that liposuction alone might provide such early improvement, the volume of aspirated fat did not significantly influence arm diameter reduction or ptosis improvement. These findings suggest that argon plasma enhances the early shrinkage observed, with the subsequent slight improvement after 30 days attributed to the steady retraction process because of fibrosis and normal healing.

Regression analysis revealed that greater preoperative tissue thickness was correlated with greater diameter and thickness reductions at 6 months (*P* = .0017 and *P* < .0001, respectively). These findings suggest that patients with greater baseline thickness may experience more pronounced tightening following treatment. Furthermore, patients with greater preoperative ptosis (larger BF-Edge distance) exhibited more significant shrinkage over time (*P* < .0001). These findings support the notion that argon plasma is particularly effective in cases of greater skin laxity, potentially offering an advantage over traditional liposuction in such patients.

Skin elasticity was assessed through Cutometer (Courage + Khazaka electronic GmbH) measurements, which revealed progressive improvements in skin firmness and elasticity (R0, R2, R5, and R7 values). Significant reductions in the R0, R2, R5, and R7 values were observed after 30 days, with stabilization thereafter.

- R0 (skin distensibility) measures the ability of the skin to deform under negative pressure. A reduction in R0 indicates firmer skin with improved tightness.
- R2 (overall elasticity) represents the skin's ability to return to its original state after deformation. A decrease in R2 suggests increased elasticity and improved rebound after stretching.
- R5 (net elasticity) focuses on the skin's pure elastic component, excluding viscous deformation. A decrease in R5 suggests enhanced skin elasticity and resistance to sagging.
- R7 (biological elasticity) measures how much the skin recovers compared with total deformation. A lower R7 value indicates improved skin firmness and resistance to external forces.

These findings indicate that argon plasma may contribute to enhanced dermal remodeling, leading to improved skin firmness and biological recovery posttreatment. Additionally, our Cutometer (Courage + Khazaka electronic GmbH) analysis supports the variability of results depending on the patient’s skin elasticity. Specifically, higher preoperative R5 and R7 values were correlated with slightly better ptosis reduction at 6 months.

One of the primary objectives of this study was to assess safety. The success of skin retraction depends on collagen shrinking, which occurs when collagen is heated through resistance to an electric current in the dermis and subcutaneous tissue. The main challenge is that the threshold for skin burns is lower than the temperature required for subdermal and subcutaneous collagen retraction. At 50°C, cell death occurs in 6 min, whereas at 60°C, cell death is instantaneous. Between 60°C and 100°C, the process leads to protein denaturation, coagulation, and subsequent collagen reorganization, along with desiccation as the cells lose water. Above 100°C, vaporization occurs, and at 200°C, carbonization is observed.^18^

Renuvion (Apyx, Clearwater, FL) is the most widely used plasma-driven device and uses helium as the conductor gas. Argon plasma employs a similar technology but uses a different gas. Helium molecules are smaller than argon molecules, leading to greater tissue diffusion. This could provide a theoretical advantage for argon over helium by reducing potential complications, such as pneumoperitoneum, pneumothorax, or subcutaneous emphysema.

Unlike other RFAL systems, such as BodyTite,^19^ the argon plasma probe lacks internal temperature monitoring. However, the target and mechanism of action are entirely different. BodyTite (Invasix Corp.) heats the tissue and dermis, whereas argon plasma targets the septa. Paul and Mulholland demonstrated that achieving dermal retraction requires very high temperatures (80°C for 2 min) to achieve 2 mm retraction.^20^ For the septa, the required temperature was lower (69°C), but the retraction was greater (6.5 mm). Plasma technology allows rapid cooling of targeted tissue (≤0.75 s) through conductive heat transfer.^21^ In our study, the cutoff point was 5 min, and although temperatures remained elevated postoperatively, there was a trend toward normalization, but temperatures were always well below the burn threshold.

The safety profile of APAL was favorable, with no major complications reported. Minor seroma formation (16.7%) occurred in a small subset of cases, with only 1 persisting beyond 1 month. This rate is comparable to or even lower than that reported with other skin-tightening technologies. Transient dysesthesia was noted in the early postoperative period but resolved spontaneously. One case of mild fibrosis at 6 months underscores the importance of proper energy application and postoperative management to minimize adverse effects.

Patient satisfaction, measured through the ARM-Q questionnaire, was significantly high. The only item that showed no significant improvement was “How smooth (no irregularities) do the arms look?” In patients with more pronounced skin laxity, the shrinkage effect produced by the procedure occasionally resulted in noticeable and unesthetic wrinkling. We believe this outcome is primarily associated with volume deflation caused by liposuction, and that the severity of wrinkling highlights the potency of argon plasma in inducing skin contraction. Moving forward, it may be necessary to modify the surgical approach—potentially adopting a less aggressive liposuction technique—to allow for adequate skin retraction without leading to dermal collapse.

The main limitations of this study include the sample size and heterogeneity, as some patients had greater volume, whereas others exhibited more skin flaccidity. Additionally, no control group was performed, which would be the most effective way to isolate the contribution of argon plasma to the outcomes.

The next steps in our research include a comparison with liposuction-only procedures and a longer follow-up period of 12 months. Additionally, the surgical technique may need to be reconsidered—more conservative liposuction might be necessary to prevent the unesthetic wrinkling observed in patients with predominant skin laxity.

Nonetheless, our findings suggest that APAL may be a safe and effective modality for arm contouring. The rapid onset and stabilization of results make it a compelling alternative to more invasive procedures, such as brachioplasty, particularly for patients with moderate skin laxity.

## CONCLUSIONS

APAL of the arms had excellent efficacy in reducing ptosis, arm diameter, and tissue thickness while improving skin elasticity in this patient cohort. The procedure offers a minimally invasive alternative to surgical arm lifting, with a favorable safety profile and predictable outcomes. These findings support its role as an innovative technique for upper-arm contouring, particularly in patients with mild-to-moderate skin laxity.

## Supplementary Material

ojaf058_Supplementary_Data
