# Correction to: Global Insights Into Confidence and Self-Perception in Aesthetic Medicine: A 15-Country Survey Analysis

**DOI:** 10.1093/asjof/ojag124

**Published:** 2026-06-29

This is a correction to: Shannon Humphrey, Claudia Andrea Hernández, Jose Raul Montes, Je-Young Park, Daria Voropai, Katherine Warnell, Global Insights Into Confidence and Self-Perception in Aesthetic Medicine: A 15-Country Survey Analysis, *Aesthetic Surgery Journal Open Forum*, Volume 8, 2026, ojag058, https://doi.org/10.1093/asjof/ojag058

The following changes have been made to the originally published paper. The upper bound, of age “70”, has been changed to “75” throughout.

The table file originally published in the **Supplementary data** section is correspondingly updated inline with these article text changes and has been replaced in the paper.

