# Supplementary material for: Argon Plasma-Assisted Liposuction for Arm Contouring: Safety, Efficacy, and Skin Tightening Outcomes
Source: Aesthet Surg J Open Forum. 2025 Jun 16;7:ojaf058. doi: 10.1093/asjof/ojaf058 (PMC12378442; doi:10.1093/asjof/ojaf058)
Supplement: ojaf058_Supplementary_Data [file ojaf058_Supplementary_Data.zip › suppl table 1.docx]

| **Id** | **Age** | **BMI** | **Side** | **D** | **Th** | **BF-Edge** | **El-Khatib** |
| --- | --- | --- | --- | --- | --- | --- | --- |
| 1 | 45 | 23,9 | Right | 348,3 | 19 | 60,4 | 2b |
| 1 |  |  | Left | 343,1 | 20 | 56,5 | 2b |
| 2 | 53 | 23 | Right | 310,3 | 19,5 | 59 | 2B |
| 2 |  |  | Left | 312,9 | 18,5 | 55 | 2B |
| 3 | 58 | 19,3 | Right | 286,2 | 20 | 42 | 2A |
| 3 |  |  | Left | 307,3 | 21,4 | 46 | 2A |
| 4 | 51 | 24,2 | Right | 332,6 | 17,9 | 57,06 | 2B |
| 4 |  |  | Left | 344,0 | 18,8 | 67,75 | 2B |
| 5 | 48 | 25.1 | Right | 344,9 | 17,4 | 61,05 | 2b |
| 5 |  |  | Left | 355,3 | 22,4 | 57,05 | 2b |
| 6 | 58 | 24,5 | Right | 388,4 | 37,2 | 69 | 2B |
| 6 |  |  | Left | 390,0 | 36,7 | 73,5 | 2B |
| 7 | 38 | 25,6 | Right | 374,0 | 15,8 | 60,35 | 2B |
| 7 |  |  | Left | 370,1 | 14,2 | 60,57 | 2B |
| 8 | 59 | 27,3 | Right | 370,8 | 36,5 | 65,8 | 2b |
| 8 |  |  | Left | 368,0 | 36,8 | 62,62 | 2b |
| 9 | 56 | 22,8 | Right | 305,8 | 15,2 | 46,96 | 2b |
| 9 |  |  | Left | 309,4 | 12,9 | 45,95 | 2b |
| 10 | 38 | 29,7 | Right | 356,4 | 33,4 | 58,3 | 2b |
| 10 |  |  | Left | 370,3 | 29,9 | 55,8 | 2b |
| 11 | 58 | 25,8 | Right | 327,4 | 14,9 | 46,7 | 2b |
| 11 |  |  | Left | 321,6 | 18,8 | 49,27 | 2b |
| 12 | 58 | 27,3 | Right | 324,9 | 16,6 | 49,5 | 2b |
| 12 |  |  | Left | 341,7 | 13,3 | 41,5 | 2b |
| 13 | 66 | 23,2 | Right | 310,0 | 17,8 | 59,7 | 2b |
| 13 |  |  | Left | 320,0 | 20,2 | 52 | 2b |
| 14 | 60 | 22,5 | Right | 325,3 | 28,2 | 51 | 2b |
| 14 |  |  | Left | 334,8 | 24,4 | 55,78 | 2b |
| 15 | 39 | 24 | Right | 320,0 | 25,4 | 52,23 | 2b |
| 15 |  |  | Left | 320,0 | 24,5 | 44,63 | 2b |

Suppl Table 1. Case series. D: diameter of the arm; Th: thickness; BF-Edge: ptosis. All measurementes in mm.
